# A novel *CACNA1S* gene variant in a child with hypokalemic periodic paralysis: a case report and literature review

**DOI:** 10.1186/s12887-023-04326-1

**Published:** 2023-10-02

**Authors:** Wen Zhou, Peilin Zhao, Jian Gao, Yunjian Zhang

**Affiliations:** 1 Department of Pediatrics, People’s Hospital of Jinping Miao, Yao and Dai Autonomous County, Honghe Prefecture, Yunnan Province China; 2 Department of Neurology, National Children’s Medical Center, Children’s Hospital of Fudan University, 399 Wanyuan Road, Minhang District, Shanghai, 201102 China

**Keywords:** *CACNA1S*, Ca_v_1.1, Calcium channel, Hypokalemic periodic paralysis

## Abstract

**Background:**

The *CACNA1S* gene encodes the alpha 1 S-subunit of the voltage-gated calcium channel, which is primarily expressed in the skeletal muscle cells. Pathogenic variants of *CACNA1S* can cause hypokalemic periodic paralysis (HypoPP), malignant hyperthermia susceptibility, and congenital myopathy. We aimed to study the clinical and molecular features of a male child with a *CACNA1S* variant and depict the molecular sub-regional characteristics of different phenotypes associated with *CACNA1S* variants.

**Case presentation:**

We presented a case of HypoPP with recurrent muscle weakness and hypokalemia. Genetic analyses of the family members revealed that the proband had a novel c.497 C > A (p.Ala166Asp) variant of *CACNA1S*, which was inherited from his father. The diagnosis of HypoPP was established in the proband as he met the consensus diagnostic criteria. The patient and his parents were informed to avoid the classical triggers of HypoPP. The attacks of the patient are prevented by lifestyle changes and nutritional counseling. We also showed the molecular sub-regional location of the variants of *CACNA1S* which was associated with different phenotypes.

**Conclusions:**

Our results identified a new variant of *CACNA1S* and expanded the spectrum of variants associated with HypoPP. Early genetic diagnosis can help avoid diagnostic delays, perform genetic counseling, provide proper treatment, and reduce morbidity and mortality.

## Background

Hypokalemic periodic paralysis (HypoPP) is a rare skeletal muscle disorder characterized by episodic muscle weakness associated with decreased serum potassium levels [1]. The prevalence of HypoPP is thought to be approximately 1/100,000 [2]. Stunnenberg et al. reported that the minimum point prevalence rate for HypoPP was 0.53/100,000 in the Netherlands [3]. Two disease-causing genes, the calcium channel gene *CACNA1S* (OMIM 114,208) and the sodium channel gene *SCN4A* (OMIM 603,967) have been identified in HypoPP [4, 5].

It has been reported that approximately 70–80% and 10% of HypoPP could be attributed to pathogenic variants in *CACNA1S* gene and *SCN4A* gene respectively, while the etiologies of at least 10% of patients are still unknown [6]. Variants in the *CACNA1S* gene have been reported to be mainly responsible for HypoPP (OMIM# 170,400) [6]. The *CACNA1S* gene is predominantly expressed in the muscle. To date, other two main diseases, including malignant hyperthermia susceptibility (OMIM# 601,887) and congenital myopathy (OMIM# 620,246), have been reported to be associated with variants in the *CACNA1S* gene [6–10].

In this study, we report the clinical and molecular characteristics of a child with HypoPP caused by a novel variant in *CACNA1S* gene. They may help the reader direct their attention to a valuable case report of HypoPP. Our report refines the genotype-phenotype map of *CACNA1S*-related disorders and expands the mutant spectrum of the *CACNA1S* gene and makes way for further research.

## Case presentation

The Chinese patient, a seven-year-old boy, has been suffering of recurrent muscle weakness for 4 years before being admitted to the hospital. The boy was the second child born to a nonconsanguineous couple. Transient walking difficulties and paralysis episodes were examined at 3 years of age. During the episodes recorded throughout hospitalization, the child was floppy and hypotonic, with global muscle weakness that predominated in the lower limbs. Multiple attacks occurred every year without identifiable triggers, and each episode lasted for approximately several days. Neurological evaluation revealed hypotonia, hyporeflexia, and reduced motor activity during these episodes. Blood tests showed the decreased levels of potassium to 1.8–2.1 mmol/L (normal range: 3.5–5.5 mmol/L) and increased levels of creatine kinase (CK) to 211–1036 U/L (normal range: 24–190 U/L) during the episodes. The complete blood count, blood gas analysis, liver and renal function, glucose, other electrolytes, thyroid hormone, and serum aldosterone levels were unremarkable. Electrocardiogram showed sinus arrhythmia. Muscular features quickly improved following an intravenous infusion of potassium chloride. The patient was asymptomatic between the episodes. Clinical examination and serum CK and potassium levels were normal between the paralysis attacks. His father showed attacks of muscle weakness following strong physical exertion. However, serum potassium levels during attacks were not detected.

**Fig. 1:**
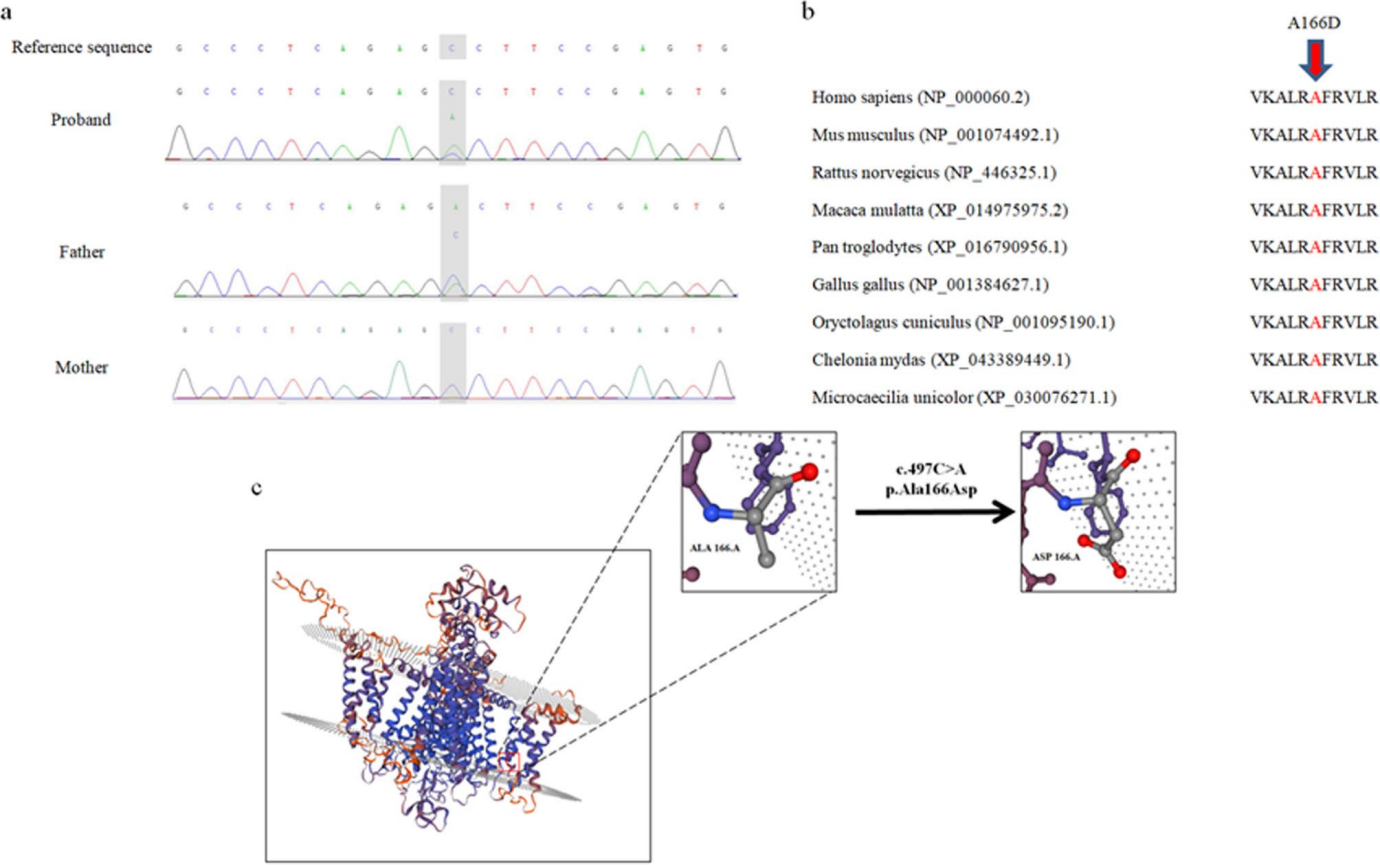
Genetic studies of the family. (a) Whole-exome sequencing and confirmation by Sanger sequencing revealed that the proband was heterozygous for c.497 C > A (p.Ala166Asp) in the *CACNA1S* gene, which was inherited from his father. (b) The A166D variant occurs at a highly conserved position in CACNA1S of different species. (c) The predicted three-dimensional structure of the Ca_v_1.1 protein denotes the effect of protein changes in A166D.

Peripheral blood samples were obtained from the patient and his parents. Genomic DNA was extracted from the blood samples. Whole-exome sequencing (WES) was performed on the proband, and Sanger sequencing was further performed to verify the candidate variants. WES was performed based on the NovaSeq 6000 Sequencing platform, the IDT XGen Exome Research Panel was used to capture libraries, and paired-end clean reads were used for comparison with the human reference genome (GRCh38/hg38). Variations were annotated using ANNOVAR and picked up with a minor allele frequency of ≤ 0.005 in the single-nucleotide polymorphism database. The pathogenicity of the variants was evaluated according to the American College of Medical Genetics and Genomics (ACMG) guidelines, as well as the clinical phenotype of the patient. “Ada” and “RF” scores were used to evaluate potential splicing variants predicted by dbscSNV. Sanger sequencing was further performed to validate the candidate variants identified by WES. A novel heterozygous variant, NM_000069.3:c.497 C > A(p.Ala166Asp), was detected in exon 4 of the *CACNA1S* gene in the proband, which was inherited from his father (Fig. 1a). This novel missense variant has not been previously reported in the gnomAD database. It was predicted to be pathogenic by using PolyPhen-2, SIFT, and MutationTaster and was classified as likely pathogenic according to ACMG guideline. The REVEL score is 0.932. The amino acid residues of the missense variant are highly conserved in various species (Fig. 1b). The 3D models of Ca_v_1.1 protein were further visualized. The encoded amino acid sequence at position 166 changed from hydrophobic alanine to hydrophilic aspartic acid owing to the c.497 C > A variant (Fig. 1c).

**Fig. 2:**
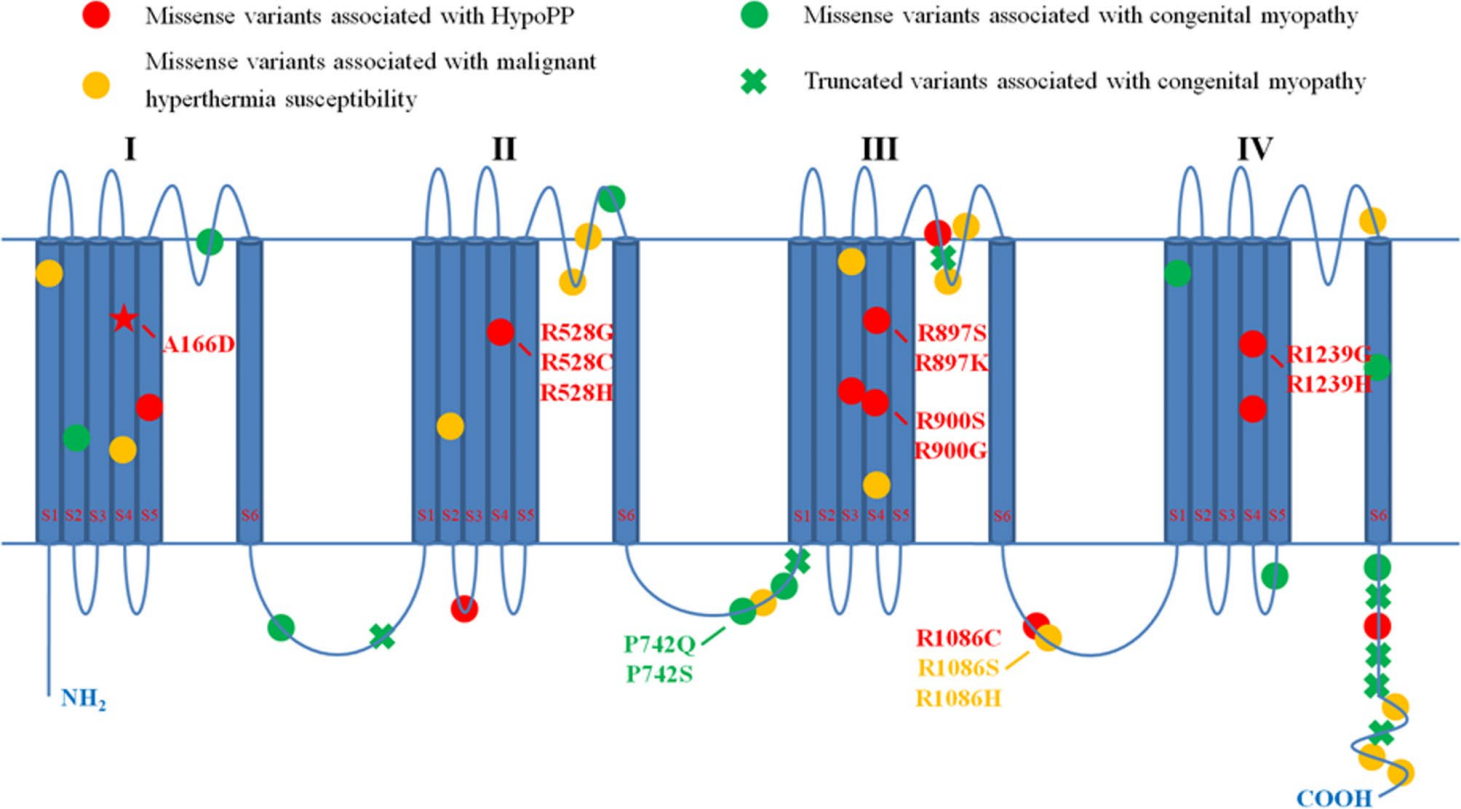
Schematic of the Ca_v_1.1 protein showing pathogenic variants associated with different phenotypes Shown are the locations of pathogenic or likely pathogenic variants of *CACNA1S* gene. Ca_v_1.1 is composed of four repeated domains (DI–DIV), each containing six transmembrane segments (S1–S6), with S1–S4 representing the voltage-sensor domains and S5–S6 forming pore domains. Variants associated with HypoPP are indicated by red circles, whereas variants identified in individuals with malignant hyperthermia susceptibility are indicated by orange circles. The green circles denote missense variants identified in patients with congenital myopathy, whereas the green crosses represent truncated variants associated with congenital myopathy. The red pentagram indicates the variant in our patient

**Fig. 3:**
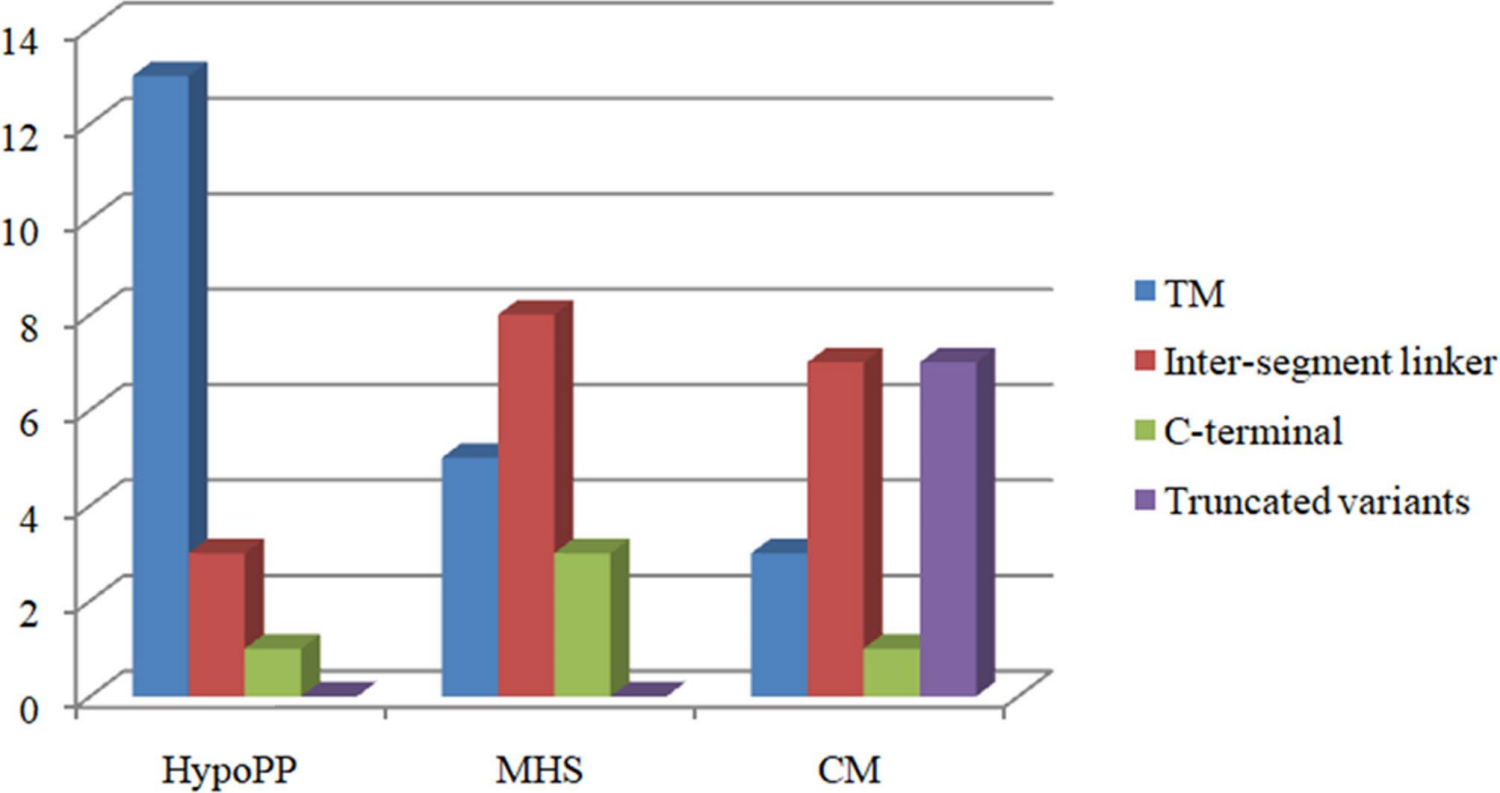
Phenotype-genotype comparison by types of *CACNA1S* gene variants. Truncated variants are grouped in a unique category; missense variants are grouped by localization of the amino-acid substitution. HypoPP: hypokalemic periodic paralysis; MHS: malignant hyperthermia susceptibility; CM: congenital myopathy; TM: transmembrane

As shown in Fig. 2, the variant of the patient was located in a new region of the Ca_v_1.1, the S4 voltage-sensor transmembrane segment of domain I, a domain that had never been associated with HypoPP. To evaluate the genotype-phenotype correlation, we systematically reviewed *CACNA1S* gene variants in the PubMed database until May 2023 with detailed phenotypes [1, 3, 7–9, 11–19]. We demonstrate that variants of HypoPP appear to be preferentially clustered in the S4 transmembrane segments. Variants located in the inter-segment linker of S5–S6 of DII–DIV and in the C-terminal were more likely to be associated with malignant hyperthermia susceptibility. Individuals carrying *CACNA1S* truncated variants were more likely to be diagnosed with congenital myopathy (Figs. 2 and 3).

After excluding endocrine and renal causes of the hypokalemic episodes, based on the typical clinical manifestations of the recurrence of flaccid paralysis episodes with a decreased serum potassium level, a response to potassium supplementation, and the genetic finding of a novel variant of *CACNA1S* gene, the patient was diagnosed with hypokalemic periodic paralysis. Subsequently, the boy was advised to take oral potassium supplementation.

Attacks of weakness may be triggered mainly by exhaustion, high-carbohydrate and sodium meals, alcohol consumption, cold exposure, stress/excitement/fear, use of corticosteroids, and anesthetic procedures. The patient was instructed to avoid these classical triggers. He also received nutritional and psychological counseling and guidance on lifestyle changes to avoid episodes of attacks. The patient did not receive acetazolamide or potassium-sparing diuretic therapy. Currently, attacks are prevented by lifestyle changes and nutritional counseling and clinical examination and potassium levels were normal between the episodes.

## Discussion and conclusions

Here, we report a patient with a novel heterozygous missense variant p.Ala166Asp of *CACNA1S* gene. In addition to recurrent episodic attacks of flaccid paralysis with concomitant hypokalemia, the proband was diagnosed with HypoPP, which is a rare autosomal dominant neuromuscular disorder.

*CACNA1S* gene plays an important role in Ca^2+^-mediated excitation-contraction coupling. The *CACNA1S* gene is located on chromosome 1q32.1, which has 44 exons, encodes 1,873 amino acids [6]. To date, several missense variants in the *CACNA1S* gene have been identified with HypoPP, most of which are located in the critical voltage-sensor S4 segment and occur at an arginine residue, including R528G, R528C, and R528H located in DIIS4, R897S, R897K, R900S, and R900G in DIIIS4, and R1239G, R1239H, and R1242S in DIVS4 [1, 4, 13, 15, 17]. Interestingly, the A166D variant identified in our patient occurs at a different alanine residue located in the S4 transmembrane segment of domain I, which had never been reported to be associated with HypoPP. In this case, the encoded amino acid sequence changed from hydrophobic alanine to hydrophilic aspartic acid at position 166. A schematic diagram of predicted Ca_v_1.1 wild-type and variant protein structures, modeled on the 3D structure, indicates that the variant may lead to dysregulation of this protein function. Therefore, these results clearly show that dysfunction of the S4 segment of domain I can also be responsible for HypoPP, and variants of HypoPP appear to be preferentially clustered in domains of voltage-sensor S4 of DI–DIV.

CACNA1S is composed of four homologous transmembrane domains (DI–DIV), each containing six segments spanning the membrane (S1–S6) [6]. We depicted the genotypes and molecular sub-regional characteristics of different phenotypes associated with *CACNA1S* gene variants. In this study, we demonstrated that variants associated with HypoPP appear to preferentially cluster in S4 of the transmembrane domains. Variants associated with malignant hyperthermia susceptibility were more likely to be located in the inter-segment linker of S5–S6 of DII–DIV and in the C-terminal. Individuals carrying *CACNA1S* truncated variants were more likely to be diagnosed with congenital myopathy. Our study showed that the molecular sub-regional location of the variants of *CACNA1S* gene was associated with different phenotypes. However, the total number of patients with *CACNA1S* gene variants was relatively small. More cases are needed to elucidate the genotype-phenotype correlation of *CACNA1S* gene in future studies.

Our patient had recurrent attacks of muscle weakness and hypokalemia, which met the consensus diagnostic criteria for HypoPP. Possible life-threatening elements of paralytic attacks include cardiac dysrhythmia, acute respiratory insufficiency, and occurrence in a hostile environment, such as drowning in a swimming pool when paralytic attacks are triggered. Kil and Kim reported a boy who presented with a severe respiratory phenotype and hypokalemic periodic paralysis caused by a heterozygous Arg528Gly mutation in the *CACNA1S* gene [17]. Cardiac dysrhythmia and acute respiratory insufficiency were not observed in our patient. It was reported that relative incomplete penetrance of this disorder was noted [2]. Our patient’s relatively milder phenotypes suggest the incomplete penetrance of this case.

The treatment principles for HypoPP include providing potassium supplementation for attacks, avoiding precipitating factors, and preventive treatment for paralytic attacks [6]. Carbonic anhydrase inhibitors, particularly acetazolamide and dichlorphenamide, are generally considered effective preventative treatments for paralytic attacks [2]. However, in patients with *SCN4A* gene variants acetazolamide use may exacerbate symptoms [2]. It was reported that various pathogenic mutations of *SCN4A* gene can cause different phenotypes including nondystrophic myotonias (due to gain of channel function) and periodic paralysis (due to inactivation of the channel) [20]. Acetazolamide may be a good treatment option for patients with nondystrophic myotonias, [20] while sodium channel inactivation may be associated with worsening of HypoPP with acetazolamide. [21] If carbonic anhydrase inhibitors are invalid or not tolerated, potassium sparing diuretics include triamterene, spironolactone, and eplerenone could be used as alternatives [22]. The patient in our study did not receive acetazolamide or potassium-sparing diuretic therapy, and the attacks were prevented by lifestyle changes and nutritional counseling.

HypoPP is inherited in an autosomal dominant manner [2]. The proband’s offspring are at 50% risk of inheriting the pathogenic variant. Therefore, prenatal testing may be advisable. Furthermore, it is important to do genetic testing of the family members, because those with *CACNA1S* gene variants may have a higher risk of anesthetic complications, such as malignant hyperthermia and severe paralysis [7]. Therefore, early genetic diagnosis is critical to avoid diagnostic delays, perform genetic counseling, take proper treatment, and reduce morbidity and mortality. This study also opens new avenues for genotype-phenotype correlations.

In summary, we described an additional case of HypoPP and identified a novel variant of *CACNA1S* gene. The molecular sub-regional location of the variants of *CACNA1S* gene may be associated with different phenotypes. Our report may provide new perspectives for understanding *CACNA1S* genotype-phenotype correlations.

## Data Availability

Data on patient and case details are available from the corresponding author on reasonable request.

## Abbreviations

HypoPP: Hypokalemic periodic paralysis.
CACNA1S: Calcium voltage-gated channel subunit alpha1 S.
SCN4A: Sodium voltage-gated channel alpha subunit 4.
WES: Whole-exome sequencing.
